# MicroRNA-148b is frequently down-regulated in gastric cancer and acts as a tumor suppressor by inhibiting cell proliferation

**DOI:** 10.1186/1476-4598-10-1

**Published:** 2011-01-04

**Authors:** Yong-Xi Song, Zhen-Yu Yue, Zhen-Ning Wang, Ying-Ying Xu, Yang Luo, Hui-Mian Xu, Xue Zhang, Li Jiang, Cheng-Zhong Xing, Yong Zhang

**Affiliations:** 1 Department of Surgical Oncology and General Surgery, First Hospital of China Medical University, 155 North Nanjing Street, Heping District, Shenyang City 110001, China; 2 The Research Center for Medical Genomics and MOH Key Laboratory of Cell Biology, China Medical University, 155 North Nanjing Street, Heping District, Shenyang City 110001, China

## Abstract

**Background:**

MicroRNAs (miRNAs) are involved in cancer development and progression, acting as tumor suppressors or oncogenes. Our previous studies have revealed that miR-148a and miR-152 are significantly down-regulated in gastrointestinal cancers. Interestingly, miR-148b has the same "seed sequences" as miR-148a and miR-152. Although aberrant expression of miR-148b has been observed in several types of cancer, its pathophysiologic role and relevance to tumorigenesis are still largely unknown. The purpose of this study was to elucidate the molecular mechanisms by which miR-148b acts as a tumor suppressor in gastric cancer.

**Results:**

We showed significant down-regulation of miR-148b in 106 gastric cancer tissues and four gastric cancer cell lines, compared with their non-tumor counterparts by real-time RT-PCR. *In situ *hybridization of ten cases confirmed an overt decrease in the level of miR-148b in gastric cancer tissues. Moreover, the expression of miR-148b was demonstrated to be associated with tumor size (P = 0.027) by a Mann-Whitney U test. We also found that miR-148b could inhibit cell proliferation *in vitro *by MTT assay, growth curves and an anchorage-independent growth assay in MGC-803, SGC-7901, BGC-823 and AGS cells. An experiment in nude mice revealed that miR-148b could suppress tumorigenicity *in vivo*. Using a luciferase activity assay and western blot, CCKBR was identified as a target of miR-148b in cells. Moreover, an obvious inverse correlation was observed between the expression of CCKBR protein and miR-148b in 49 pairs of tissues (P = 0.002, Spearman's correlation).

**Conclusions:**

These findings provide important evidence that miR-148b targets CCKBR and is significant in suppressing gastric cancer cell growth. Maybe miR-148b would become a potential biomarker and therapeutic target against gastric cancer.

## Introduction

Gastric cancer is the second leading cause of cancer-related mortality worldwide [1]. As with other cancers, the development of gastric cancer is a multistep process with the accumulation of genetic and epigenetic changes. Many molecular mechanisms have been revealed, but the role of microRNAs (miRNAs) remains to be elucidated in gastric cancer.

MiRNAs are a recently discovered class of naturally occurring small non-coding RNAs involved in the regulation of gene expression by targeting mRNAs for translational repression or cleavage [2,3]. Fifty percent of miRNAs are located in the chromosomal regions known to be frequently amplified or deleted in human cancer cells [4]. Growing evidence indicates that these short RNAs of 19 to 25 nucleotides play key roles in a wide variety of biological processes including cell fate specification, proliferation, cell death, and energy metabolism [5-8]. Furthermore, an increasing number of miRNAs have been shown to be involved in cancer development and progression [9-13].

Our previous studies have revealed that miR-148a and miR-152 are down-regulated in gastrointestinal cancers [14]. Moreover, bioinformatics shows that miR-148b has the same "seed sequences" as miR-148a and miR-152, however, its pathophysiologic role and relevance to tumorigenesis are still largely unknown. MiR-148b is located at chromosome 12q13 and recent studies have found it is down-regulated in oral, pancreatic, colon and gastric cancer tissues using microarray analysis [15-18]. Moreover, some studies revealed that miR-148b may play a critical role in osteogenesis [19] and may influence the polymorphism of HLA-G [20]. Furthermore, Zhao et al. found miR-148b may play a role in metastasis related to hepatocellular carcinoma [21]. Interestingly, a recent study showed that the coding sequence of DNA methyltransferase 3b (Dnmt3b) mediated regulation by the miR-148 family (miR-148a and miR-148b) [22]. However, most studies addressing the molecular mechanism of miR-148b in cellular transformation and tumorigenesis are still in an early stage. Thus, the identification of the function of miR-148b is critical to understanding the role of miR-148b in cancer development.

In this study, we analyzed miR-148b expression in human gastric cancer tissues and their matched non-tumor adjacent tissues by both qRT-PCR assay and *in situ *hybridization. Further investigation revealed that in gastric cancer cell lines, miR-148b functioned as a tumor suppressor and overexpression of miR-148b could inhibit cell proliferation *in vitro *and *in vivo*. Subsequent experiments confirmed that the cholecystokinin-B receptor (CCKBR) was a target of miR-148b and was down-regulated by miR-148b at the translational level. Our findings will help to elucidate the functions of miRNAs and their roles in tumorigenesis.

## Materials and methods

### Human Tissue Samples

One hundred and six pairs of human gastric tissue samples were obtained from patients who underwent surgical resection at the First Hospital of China Medical University between 2007 and 2008 and were diagnosed with gastric cancer based on histopathological evaluation. The matched non-tumor adjacent tissue was obtained from a segment of the resected specimens that was the farthest from the tumor (> 5 cm). The samples were snap-frozen in liquid nitrogen and stored at -80°C. No local or systemic treatment was conducted in these patients before the operation. One section of each sample was stained with hematoxylin-eosin (H&E) and was used for histopathological evaluation. The tumor histological grade was assessed according to World Health Organization criteria and was staged using the TNM staging of the International Union Against Cancer (UICC)/American Joint Committee on Cancer (AJCC) system (2002). The study was approved by the Research Ethics Committee of China Medical University, China. Informed consent was obtained from all patients.

### Cell lines and culture conditions

Human gastric cancer cell lines MGC-803, BGC-823, SGC-7901 and AGS and the normal gastric epithelium cell line (GES-1) were purchased from the Institute of Biochemistry and Cell Biology at the Chinese Academy of Sciences (Shanghai, China). MGC-803, SGC-7901 and BGC-823 were cultured in RPMI 1640 medium (Invitrogen). AGS was cultured in F12 medium (Invitrogen). GES-1 was cultured in Dulbecco's Modified Eagle medium (Invitrogen). They were all supplemented with 10% fetal boving serum (Invitrogen) at 37°C and 5% CO_2_.

### RNA isolation and real-time RT-PCR

Total RNA from the specimens and cultured cells, with efficient recovery of small RNAs, was isolated using the miRVana RNA isolation kit according to the manufacturer's instructions (Ambion). Poly(A) tail was added to the RNA using a Poly(A) Tailing Kit according to the manufacturer's instructions (Ambion). The first-strand cDNA was synthesized using the SuperScript^® ^III First-Strand Synthesis System according to the manufacturer's instructions (Invitrogen). Quantitative PCR was done using EXPRESS SYBR^® ^GreenER™ qPCR Supermix Universal (Invitrogen) and was performed in a Real-time PCR System Rotor-Gene 6000 (Qiagen). The expression of miRNAs was calculated relative to U6 small nuclear RNA. Changes in expression were calculated using the ΔΔC_t _method [23]. The relative expression ratio of miR-148b was presented as the fold change normalized to an endogenous reference (U6 RNA) and relative to the nontumorous controls (normal tissues and normal cell line). Therefore, the value of the relative expression ratio <1.0 was considered as low expression in cancer relative to the nontumorous control and the ratio >1.0 was considered as high expression. Primers used for RT-PCR are indicated in Additional file 1 Table S1.

### *In situ *hybridization

*In situ *detection of miR-148b was performed on paraffin sections using a DIG-labeled miRCURY™ Detection probe according to the manufacturer's instructions (Exiqon). Ten cases of gastric cancer were selected. The sections were deparaffinized and deproteinated, which was followed by prehybridization, hybridization [hybridization temperature = Tm probe - 21°C], stringency washing and immunological detection. The sections were then exposed to a streptavidin-peroxidase reaction system and developed with 3,3'-diaminobenzidine (DAB). Slides were counterstained with hematoxylin and analyzed with a Nikon 80i microscope and Nikon NIS-Elements F 2.3 software (Nikon).

### RNA oligoribonucleotides and cell transfection

MiR-148b mimics was a RNA duplex (Additional file 1, Table S2) designed as described previously [24]. The negative control (NC) RNA duplex (Additional file 1, Table S2) was nonhomologous to any human genome sequences. For growth curves, an anchorage-independent growth assay *in vitro *and tumorigenicity assay *in vivo*, all pyrimidine nucleotides in the miR-148b or NC duplex were substituted by their 2-O-methyl analogues to improve RNA stability. The anti-miR-148b, with sequence of 5'-ACAAAGUUCUGUGAUGCACUGA-3', was a 2'-O-methyl-modified oligoribonucleotide designed as an inhibitor of miR-148b. The anti-NC, with a sequence of 5'-CAGUACUUUUGUGUAGUACAA-3', was used as a negative control for anti-miR-148b in the antagonism experiment. The specific siRNA sequence 5'-AAGCGCGTGGTGCGAATGTTG-3' resides in exon 5 of the human CCKBR gene (Genbank accession no. NM_176875). The control siRNA sequence, 5'-AAGCTTCATAAGGCGCATAGC-3' is located on chromosome 11 of the mouse and has no homology with the human genome by BLAST comparison. All the RNA oligoribonucleotides were purchased from Genepharma (Shanghai, China).

Transfection was performed with lipofectamine 2000 Reagent (Invitrogen) following the manufacturer's protocol. A final concentration of 50 nM of RNA mimics or 200 nM of inhibitor or 100 nM of siRNA and their respective negative controls were used for each transfection in proliferation, cell cycle, apoptosis and animal experiments. Moreover, a blank control was set up for each transfection without plasmids. Transfection efficiency was monitored by qRT-PCR or western blot.

### Cell proliferation assay

#### MTT proliferation assay

The capacity for cellular proliferation was measured with a 3-(4,5-dimethylthiazol-2-yl)-2,5-diphenyltetrazolium bromide (MTT) assay. Twenty-four hours after RNA transfection, cells (approximately 8 × 10^3^) were seeded into 96-well culture plates for 24 h, 48 h, 72 h and 96 h. The cells were then incubated with 20 μL of MTT (5 mg/mL) for 4 h at 37°C and 150 μL of DMSO was added to solubilize the crystals for 20 min at room temperature. The optical density was determined with a spectrophotometer (Multiskan MK3 [Thermo]) at a wavelength of 490 nm.

#### Growth curves

Twenty-four hours after RNA transfection, equal numbers (approximately 4 × 10^4^) of cells were seeded into six-well plates. The cells were harvested and counted by trypan blue exclusion method every day after seeding.

### Anchorage-independent growth assay

Twenty-four hours after RNA transfection, SGC-7901 cells (2 × 10^2^) were suspended in 2 mL of 0.3% agarose with RPMI 1640 medium containing 10% FBS and plated into six-well plates on top of an existing layer of 0.6% agarose prepared with the same medium. The plates were incubated at 37°C in a 5% CO_2 _incubator. After four weeks, cell colonies were fixed with methanol and stained with 0.1% crystal violet for 10 min. Then, the colonies were captured with Olympus SZX12 and Qcapture Pro software (Olympus). Cell colonies >0.1 mm in diameter were counted under a microscope.

### Cell cycle analysis

For cell cycle analysis, 48 h after transfection, the adhered cells were obtained by trypsinization and pooled with the floating cells and centrifuged at 1000 rpm for 5 min. Propidium iodide (0.05 mg/ml, Sigma) and RNAseA (0.1 mg/ml, Sigma) were added to the cells and samples were analyzed 30 min after staining with the use of flow cytometry -BD FACSCalibur (BD) and CellQuest software.

### Apoptosis assay

For apoptosis assays, floating and adherent cells were harvested 24 h or 48 h after transfection, and then combined and washed with PBS. Annexin-V in combination with propidium iodide (KeyGen) was added to the cells and samples were analyzed within 30 min after staining. Quantification of fluorescence was done by flow cytometry as described above.

### Tumorigenicity assays in nude mice

Six-week-old female BALB/c athymic nude mice were subcutaneously injected in the right armpit region with 1.5 × 10^6 ^cells in 0.15 mL of PBS. Three groups of mice (n = 11) were tested. Group 1 (miR-148b mimics) was injected with MGC-803 cells transfected with miR-148b mimics; group 2 (NC) was injected with MGC-803 cells transfected with NC; and group 3 (MGC-803) was injected with MGC-803 cells alone. The tumor size was measured every 2 or 3 days with calipers. The tumor volume was calculated with the formula: (L × W^2^)/2, where L is the length and W is the width of the tumor. After the mice were killed at four weeks, the weights of the tumors were measured. All experimental procedures involving animals were in accordance with the Guide for the Care and Use of Laboratory Animals (NIH publication no. 80-23, revised 1996) and were performed according to the institutional ethical guidelines for animal experiments.

### Bioinformatics method

The miRNA targets predicted by computer-aided algorithms were obtained from PicTar (http://pictar.mdc-berlin.de/[25]), TargetScan (http://www.targetscan.org[26]) and miRBase Targets (http://www.mirbase.org/[27]). Then, the overlap of these results was further studied by Expression Analysis Systematic Explorer (EASE) analysis based on the Gene ontology database and KEGG pathway database [28].

### Vector construction

Luciferase reporters were generated based on the firefly luciferase expressing vector pGL3-control (Promega). To construct pGL3-CCKBR-3'UTR, a partial 3'UTR of the CCKBR segment of human CCKBR mRNA (1568-2044 nt, Genbank accession no. NM_176875) containing the putative miR-148b binding sites was amplified and cloned into the vector pGL3-control. We then used the same method to construct pGL3-DNMT1-3'UTR (5080-5399 nt, Genbank accession no. NM_001130823), pGL3-WNT10B-3'UTR (1574-1844 nt, Genbank accession no. NM_003394), pGL3-NOG-3'UTR (1299-1457 nt, Genbank accession no. NM_005450) and pGL3- ROBO1-3'UTR (5903-6340 nt, Genbank accession no. NM_002941). We constructed another two luciferase reporters. One was pGL3-CCKBR-3'UTR-conserved, which contains a putative miR-148b binding site in a conserved region of 3'UTR (1904-2044 nt). The other was pGL3-CCKBR-3'UTR-poorly conserved, which contains a putative miR-148b binding site in a poorly conserved region of 3'UTR (1568-1753 nt). In addition, we also constructed a luciferase reporter that had a complete complementary sequence to miR-148b as a positive control (PC). Primers used for vector construction are indicated in Additional file 1 Table S3.

### Luciferase activity assay

Cells were seeded in 24-well plates at 5 × 10^4 ^cells per well the day before transfection. 400 ng of luciferase reporter, 40 pmol (miR-148b mimics or NC) and 40 ng of pRL-TK were added in every well. Cells were collected 48 h after transfection and analyzed using the Dual-Luciferase Reporter Assay System (Promega) and Centro LB 960 (Berthold).

### Protein extraction and western blot

Total protein from the specimens and cultured cells was extracted using the total protein extraction kit according to the manufacturer's instructions (KeyGen). Proteins were separated by 8% SDS polyacrylamide gels and electrophoretically transferred to polyvinylidene difluoride membranes (Millipore). Membranes were blocked in 5% non-fat milk in TBS with 0.05% Tween-20 (TBST) at room temperature for 1 h. Antibodies directed against CCKBR (1:200, Abcam) and β-actin (1:5000, Sigma) were used. The proteins were visualized with an ECL kit (Pierce) and MF-Chemi BIS 3.2 Pro (Micro Photonics) with GelCapture Version software. The intensity of protein fragments was quantified using FluorChem 2.01 software (Alpha Innotech). CCKBR protein levels in cancer tissues were presented as fold change normalized to an endogenous reference (β-actin protein) and relative to the matched non-tumor adjacent tissues. Therefore, the fold change of CCKBR protein <1.0 was considered as low expression, whereas the fold change of CCKBR protein >1.0 was regarded as high expression.

### Statistical analysis

Data are presented as mean ± SD from at least three separate experiments. Statistical analysis was performed with Student's t-test, non-parametric test (Mann-Whitney U test between 2 groups and Kruskall-Wallis test for 3 or more groups). The statistical significance of correlations between the expression of miR-148b and CCKBR protein were calculated by a chi-square test and Spearman's rank correlation. Statistical analysis was performed using SPSS 16.0 computer software. Differences were considered statistically significant at P < 0.05.

## Results

### Expression of miR-148b and its correlation with clinicopathological characteristics of gastric cancer

**Figure 1:**
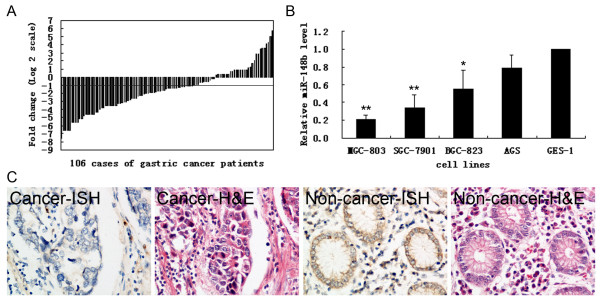
**The expression of miR-148b in tissues and cell lines**. **(A)**, MiR-148b was detected in 106 gastric cancer patients by qRT-PCR. Data were presented as log2 of fold change of gastric cancer relative to non-tumor adjacent tissues. The cases below the line (log2 = -1) revealed >50% reduction in the miR-148b level. **(B)**, The relative level of miR-148b in gastric cancer cell lines (MGC-803, SGC-7901, BGC-823, AGS) relative to normal gastric epithelial cell line (GES-1). **(C)**, H & E staining and detection of miR-148b by *in situ *hybridization in serial sections from gastric cancer tissue and its matched non-tumor adjacent tissue. Images are overlay images with brown color representing miR-148b expression. Data are presented as mean ± SD from at least three separate experiments. *, P < 0.05; **, P < 0.01.

Using a qRT-PCR method, miR-148b was detected in all 106 (100%) pairs of gastric cancer tissues and their matched non-tumor adjacent tissues, as well as the gastric cell lines. Among 106 patients with gastric cancer, 66 of 106 (62.26%) cases revealed >50% reduction in the miR-148b level relative to their matched non-tumor adjacent tissues (Figure 1A). In cell lines, we investigated the expression level of miR-148b in gastric cancer cell lines (MGC-803, SGC-7901, BGC-823 and AGS) relative to the normal gastric epithelial cell line (GES-1). Compared with GES-1, miR-148b was down-regulated with different expression levels in MGC-803 (0.21 ± 0.04-fold), SGC-7901 (0.34 ± 0.15-fold), BGC-823 (0.55 ± 0.21-fold) and AGS (0.79 ± 0.14-fold; Figure 1B). Furthermore, with *in situ *hybridization of ten cases, miR-148b was detected both in the normal epithelial cells and in the carcinoma cells (Figure 1C). Results confirmed an overt decrease in the level of miR-148b compared to their matched non-tumor adjacent tissues (Figure 1C and Additional file 2, Figure S1).

We then studied the correlation between miR-148b expression and clinicopathological characteristics of gastric cancer. The Mann-Whitney U test revealed that the expression levels of miR-148b were associated with tumor size (P = 0.027) in gastric cancer patients (Table 1). The patients with low expression of miR-148b tended to have larger tumor sizes (≥6 cm).

**Table 1:** Associations between the expression of miR-148b with clinicopathological features in 106 patients with gastric cancer

	n	miR-148b ^a^
**Sex**		
Male	80	0.28(0.08-1.26)
Female	26	0.47(0.25-1.24)
**P**		**0.272**
**Age (years)**		
≤65	65	0.41(0.10-1.24)
> 65	41	0.27(0.08-1.26)
**P**		**0.500**
**Tumor size (cm)**		
< 6	73	0.46(0.13-1.47)
≥6	33	0.23(0.05-0.43)
**P**		0.027*
**Macroscopic type**		
Early stage	3	1.82(1.67-1.85)
Borrmann I+II	9	0.50(0.26-1.01)
Borrmann III+IV	94	0.31(0.08-1.16)
**P**		**0.098**
**Histologic grade**		
Good	23	0.31(0.10-0.61)
Poor	83	0.41(0.08-1.28)
**P**		**0.626**
**pT stage**		
T1+T2	51	0.39(0.09-1.27)
T3+T4	55	0.36(0.08-1.23)
**P**		**0. 985**
**pN stage**		
N0	25	0.28(0.08-0.69)
N1	34	0.39(0.08-0.94)
N2	29	0.47(0.20-1.70)
N3	18	0.17(0.05-0.64)
**P**		**0.171**
**pTNM stage**		
I	20	0.35(0.09-0.66)
II	22	0.33(0.08-0.94)
III	39	0.43(0.17-1.27)
IV	25	0.27(0.06-3.13)
**P**		**0.821**
**Invasion into lymphatic vessels**		
Negative	77	0.41(0.10-1.30)
Positive	29	0.27(0.06-1.00)
**P**		**0.346**

^a ^Median of relative expression, with 25th-75th percentile in parenthesis.

*Indicated statistical significance (P < 0.05).

### MiR-148b inhibits cell proliferation *in vitro*

The significant reduction of miR-148b expression in gastric cancer samples prompted us to explore the possible biological significance of miR-148b in tumorigenesis. As an initial step, we detected expression of miR-148b by qRT-PCR 48 h after transfection of miR-148b mimics, anti-miR-148b, their respective NCs and blank controls (MGC-803). Transfection efficiency was perfect (Additional file 2 Figure S2).

**Figure 2:**
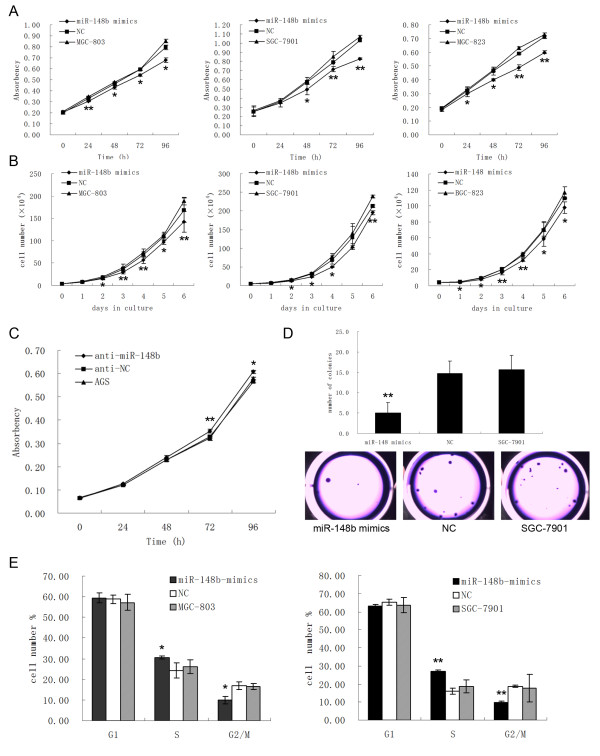
**MiR-148b inhibits cell proliferation *in vitro***. **(A)**, MTT proliferation assay in MGC-803, SGC-7901 and BGC-823. **(B)**, Growth curves by counting cell number in MGC-803, SGC-7901 and BGC-823. **(C)**, MTT proliferation assay in AGS. **(D)**, Anchorage-independent growth assay in SGC-7901 cells. The colonies were counted (top) and captured (bottom). **(E)**, The results of cell cycle analysis in MGC-803 and SGC-7901 cells. All results were reproducible in three independent experiments. *, P < 0.05; **, P < 0.01.

According to the results of the MTT assay and growth curves, we found that the cells (MGC-803, SGC-7901 and BGC-823), which were transiently transfected with miR-148b mimics, had a significant growth inhibition at different degrees (Figure 2A, B). To provide further evidence that miR-148b was indeed involved in gastric cancer cell growth, we studied the effect of the inhibitor of miR-148b in AGS cells. Proliferation of the cells transfected with anti-miR-148b was increased compared with that of the cells transfected with anti-NC and blank controls (Figure 2C).

To determine whether the inhibition of growth induced by miR-148b in cells was anchorage-independent, the cells were plated on soft agar 24 h after RNA transfection in SGC-7901 cells. After four weeks, the cells transfected with miR-148b mimics formed significantly fewer colonies on soft agar than cells transfected with NC and the blank control (Figure 2D). To further examine whether the decrease in proliferation of MGC-803, SGC-7901 and BGC-823 cells reflected a cell cycle arrest, cell cycle progression was analyzed by propidium iodide staining and flow cytometer analysis. The results revealed that MGC-803 and SGC-7901 cells transfected with miR-148b mimics had an obvious cell cycle arrest at the S-G2/M phase (Figure 2E). However, we did not get a similar result in BGC-823 cells. On the other hand, the apoptosis assay revealed that miR-148b had no effect on apoptosis in MGC-803 cells (Additional file 2, Figure S3).

### MiR-148b suppresses tumorigenicity *in vivo*

**Figure 3:**
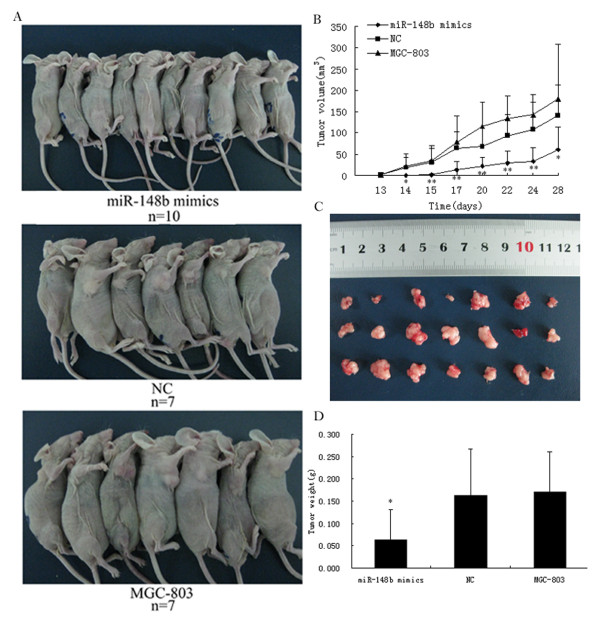
**MiR-148b suppresses tumorigenicity *in vivo***. Three groups of mice (n = 11) were tested. **(A) **, the number of alive mice and the size of tumors in three groups. **(B)**, the tumor growth curves of three groups during four weeks. **(C)**, Each tumor lump was removed from the body, and three mice with miR-148b mimics didn't form any tumors. **(D)**, the mean tumor weight of three groups at the end of the experiment. Data are presented as mean ± SD. *, P < 0.05; **, P < 0.01.

To confirm the above findings, an *in vivo *tumor model was used. MiR-148b mimics-transfected MGC-803 cells (miR-148b mimics), NC-transfected MGC-803 cells (NC) and MGC-803 cells were injected separately into three groups of nude mice (n = 11). Four weeks after injection, the group with miR-148b mimics had a lower mortality rate (9.09%) and formed substantially smaller tumors than the other 2 groups (Figure 3A). The tumor volume at the time of death in mice injected with miR-148b mimics-transfected cells was 60.49 ± 53.29 mm^3^, whereas the tumor volume of mice injected with NC or MGC-803 cells were 140.81 ± 72.21 mm^3 ^and 180.43 ± 127.89 mm^3^, respectively (Figure 3B). Moreover, the mean tumor weight at the end of the experiment was markedly lower in the group with miR-148b mimics (0.063 ± 0.068 g) compared to the NC and MGC-803 groups (0.162 ± 0.106 g and 0.17 ± 0.091 g; Figure 3C, D).

### CCKBR is a potential target of miR-148b in MGC-803, SGC-7901 and BGC-823 cells using a luciferase activity assay

**Figure 4:**
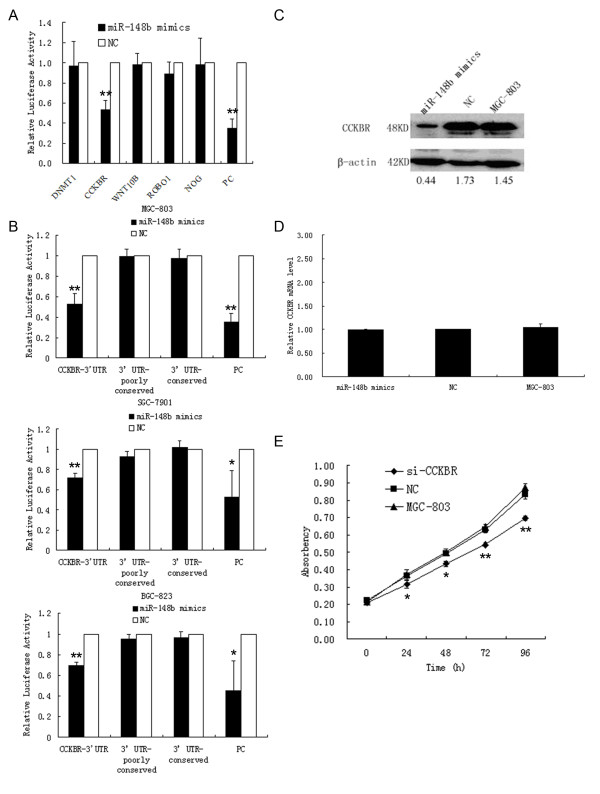
**CCKBR is a potential target of miR-148b in SGC-7901, MGC-803, BGC-823 cells**. **(A)**, analysis of luciferase activity of five possible target genes with miR-148b mimics or NC in MGC-803 cells. **(B)**, analysis of luciferase activity of pGL3-CCKBR-3'UTR, pGL3-CCKBR-3'UTR-poorly conserved, pGL3-CCKBR-3'UTR-conserved and positive control (PC) with miR-148b mimics or NC in MGC-803, SGC-7901 and BGC-823 cells. **(C&D)**, Western blot and qRT-PCR were used to monitor the expression level of CCKBR in MGC-803 cells 48 h after transfection with miR-148b mimics or NC. **(E)**, Effects of knockdown of the CCKBR gene on cell proliferation in MGC-803 cells.

Many putative miR-148b targets are predicted by various computer-aided algorithms. After Expression Analysis Systematic Explorer (EASE) analysis, eleven genes were picked out as candidate targets of miR-148b (Additional file 1 Table S4). Among the possible candidates, we further analyzed DNMT1, CCKBR, WNT10B, NOG and ROBO1, which are five genes whose functions have been associated with carcinogenesis or cancer development. Interestingly, the relative luciferase activity of these reporters was suppressed at different degrees when miR-148b mimics was cotransfected in MGC-803 cells compared with the NC (Figure 4A). However, we found that the relative luciferase activity of the pGL3-CCKBR-3'UTR reporter was significantly suppressed. These results indicate that CCKBR may be a target of miR-148b.

Then, we found that the 3'-UTR of CCKBR has two presumed sites in the conserved and poorly conserved regions that can bind with the seed region of miR-148b. So we constructed another two luciferase plasmids, pGL3-CCKBR- 3'UTR-conserved and pGL3-CCKBR-3'UTR-poorly conserved. Furthermore, we analyzed these luciferase reporters in MGC-803, SGC-7901 and BGC-823 cells. The results revealed that the relative luciferase activity of the pGL3-CCKBR-3'UTR reporter was obviously suppressed by miR-148b mimics in these cells but pGL3-CCKBR-3'UTR-conserved or pGL3-CCKBR-3'UTR-poorly conserved had no overt variation (Figure 4B).

### The inverse correlation between the expression of miR-148b and CCKBR protein in cells and tissue samples

To confirm CCKBR is a target of miR-148b, we transfected MGC-803 cells with miR-148b mimics and the NC. Forty-eight hours after transfection, we examined the CCKBR protein levels in cells by western blot. Obviously, CCKBR protein levels were suppressed by miR-148b mimics in MGC-803 cells (Figure 4C). Despite the effect of miR-148b on CCKBR protein levels, no effect on the CCKBR mRNA level was detected by qRT-PCR (Figure 4D).

In addition, we measured CCKBR protein levels in 49 pairs of the previously studied 106 gastric cancer tissues and their matched non-tumor adjacent tissues, which had already been verified as expressing miR-148b by qRT-PCR. After we quantified the protein fragments, an obvious inverse correlation was observed between the expression of CCKBR and miR-148b in tissue samples (P = 0.002, Table 2). Nevertheless, the correlation coefficient was not perfect (r = -0.436).

**Table 2:** Correlation between the expression of miR-148b and CCKBR protein in 49 gastric cancer cases

	miR-148b ^a^				Spearman's correlation	
				
	low	high	n	p	p	r
CCKBR ^b^						
low	8	6	49	0.002	0.002	-0.436
high	34	1				

^a^Number of cancers with reduced or increased levels of miR-148b relative to non-tumor adjacent tissues.

^b^Number of cancers with reduced or increased levels of CCKBR protein relative to non-tumor adjacent tissues.

### Effects of knockdown of the CCKBR gene on cell proliferation in MGC-803 cells

The results above showed that miR-148b could inhibit cell proliferation *in vitro *and suppress tumorigenicity *in vivo*. Moreover, CCKBR is a potential target of miR-148b using a luciferase activity assay. Therefore, we asked whether CCKBR has an effect on gastric cancer cell growth. To this end, the expression of the CCKBR gene was knocked down by transfection of MGC-803 cells with siRNA (Additional file 2 Figure S4). A MTT proliferation assay showed that knockdown of CCKBR gene expression significantly inhibited cell proliferation (Figure 4E).

## Discussion

Until now, miRNA expression was studied by microarrays [29], bead-based flow cytometric assay [30], northern blot [31], real-time PCR [32], *in situ *hybridization [33], modified Invader assay [34] and other techniques. However, real-time PCR has an advantage in that it is a more quantitative and more sensitive method compared with other high-throughput assays. Moreover, although altered expression levels of many miRNAs have been identified in human cancers [35], few studies have described miRNA expression associated with clinicopathological characteristics in gastric cancer. Therefore, we used real-time PCR and *in situ *hybridization to profile the expression of miR-148b in a large number of cases and clarified the relationship between miR-148b and clinicopathological characteristics in gastric cancer.

In our study, we found significant low-expression of miR-148b in gastric cancer tissues and cell lines. Ueda et al. also found this tendency in gastric cancer tissues by microarray analysis [18]. Taken together, these results suggest that reduced miR-148b is a frequent event in human gastric cancer tissues and may be involved in carcinogenesis as a tumor suppressor gene. Nevertheless, although down-regulation of miR-148b was detected in some cancers, only Zhao et al. found miR-148b may have a relationship with metastasis in hepatocellular carcinoma [21]. However, in the present study, miR-148b expression was found to be associated with tumor size (P = 0.027) in gastric cancer patients. The patients with low expression of miR-148b tended to have larger tumor sizes (≥6 cm). Moreover, tumor size has been considered as an important prognostic factor for gastric cancer patients [36]. In view of the above, we speculate that miR-148b may participate in gastric cancer progression.

Deregulated cell proliferation is a key mechanism for neoplastic progression [37]. Our MTT assay and growth curves results both indicate that miR-148b is associated with significant growth inhibition in gastric cancer cells at different degrees. This was further supported by the finding that the overexpression of miR-148b could inhibit tumor formation and growth in nude mice. Moreover, these findings also confirm the previous result of qRT-PCR. On the other hand, what is responsible for miR-148b induced inhibition of proliferation? Generally, cell cycle arrest is an important factor [38,39]. Indeed, our subsequent cell cycle analysis revealed that MGC-803 and SGC-7901 cells transfected with miR-148b mimics had an obvious cell cycle arrest at the S-G2/M phase. Nevertheless, we did not get a similar result in BGC-823 cells. Bandi et al. documented that cell cycle arrest induced by miR-15a and miR-16 depended on the expression of Rb [9]. Possibly the process of cell cycle arrest induced by miR-148b is influenced by another molecular mechanism as indirect regulation. This also indicates that miR-148b may play different roles in different cells.

The fundamental function of miRNA is to regulate targets by direct cleavage of the mRNA or by inhibition of protein synthesis, according to the degree of complementarities with the 3'UTR of targets. Perfect or nearly perfect base pairing induces target mRNA cleavage, whereas imperfect base pairing induces mainly translational silencing of the target [40]. The bioinformatics analysis has estimated that as many as 30% of human genes are miRNA targets [41]. In the present study, one of our predicted target genes is CCKBR. Moreover, we used a luciferase activity assay and western blot to confirm that CCKBR is a target of miR-148b in cells. And subsequently, an obvious inverse correlation was observed between the expression of CCKBR and miR-148b in 49 pairs of tissue samples. However, miR-148b has no effect on the CCKBR mRNA level detected by qRT-PCR. These results highlight that miR-148b interacts with CCKBR and negatively regulates its expression at the translational level.

CCKBR is widely distributed throughout the human gastrointestinal tract, pancreas, lung and some neuroendocrine tissues. The main function is to mediate the normal physiological function of gastrin. Gastrin acts as a potent cell-growth factor and has proliferative effects on various malignancies including gastric, colorectal, pancreatic, medullary thyroid cancers and small cell lung cancer, as well as tumors of the central and peripheral nervous systems through CCKBR [42-47]. In the present study, we found that knockdown of CCKBR gene expression significantly inhibited MGC-803 cell proliferation. Moreover, a recent study also found that disrupting the gastrin-CCKBR autocrine loop by neutralizing the endogenous gastrin or by knocking down CCKBR expression significantly inhibited cell proliferation in SGC-7901 cells [48]. Therefore, we propose that miR-148b may have an effect on proliferation in gastric cancer, depending on regulating the expression of CCKBR. Interestingly, luciferase reporters, pGL3-CCKBR-3'UTR-conserved and pGL3-CCKBR-3'UTR-poorly conserved, which have presumed sites, did not show a significant correlation with miR-148b. Possibly, miR-148b regulates the expression of CCKBR depending on other binding sites or three-dimensional reconstructions of 3'UTR. Furthermore, although we found an obvious inverse correlation between the expression of CCKBR and miR-148b in tissue samples, the correlation coefficient was not perfect (r = -0.436). It is worth considering whether there are other mechanisms involved in the regulation processes of miR-148b.

Lars et al. documented a direct link between miR-129 and 2 putative targets GALNT1 and SOX4 by luciferase activity assay in HEK293 cells [11]. The miR-15a and miR-16 cluster was also demonstrated as having more than one target [9,10,49]. Moreover, miR-101 regulated the expression of Mcl-1 in liver cancer cells and regulated cyclooxygenase-2 in colon cancer cells [12,50]. Duursma et al. studied the target of miR-148 in the protein coding region and found that human miR-148 represses the expression of the DNA methyltransferase 3b (Dnmt3b) gene, which is the primary mediator of establishment and maintenance of DNA methylation in mammals [22]. Therefore, miR-148b may regulate different targets in the same cells, in different cells or depending on different binding regions. Moreover, although CCKBR can be regulated by miR-148b, an indirect mechanism cannot be excluded.

The 2 nt - 8 nt of miRNA, known as the "seed region", has been suggested to be the most important recognition site [51]. As shown on the miRbase website, miR-148a, miR-148b and miR-152 have the same "seed sequences". Linsley et al. reported that, in a colon carcinoma cell line, the microRNA-16 family could regulate different targets in a coordinated fashion, including CDK6, CARD10, CDC27 and C10orf46, which act in cell cycle progression [49]. Braun et al. studied miR-192 and miR-215, which have the same "seed sequences", and showed they can both act as effectors as well as regulators of p53 [52]. Moreover, Katada et al. and our previous work studied the expression of miR-148a and miR-152 in gastric cancer tissues and found they were down-regulated compared with non-tumor adjacent tissues [53,14]. We also found CCKBR was a putative target of miR-148a and miR-152 previously [14]. Therefore, miR-148a, miR-148b and miR-152 may play the same role in gastric cancer by regulating the same targets, and the relationship among them need further investigation.

In conclusion, we showed there was significant low-expression of miR-148b in gastric cancer tissues and cell lines compared with their non-tumor counterparts. Moreover, the expression of miR-148b was found to be associated with tumor size in gastric cancer patients. Our data also suggest that miR-148b can inhibit cell proliferation *in vitro *and *in vivo*. Maybe miR-148b would become a potential biomarker and therapeutic target against gastric cancer. However, the regulation processes of miR-148b, as well as the relationship between miR-148a, miR-148b and miR-152, need further study.

## Competing interests

The authors declare that they have no competing interests.

## Authors' contributions

YXS, ZYY, ZNW, YYX and YL carried out the experimental work, LJ provided data analysis, HMX and CZX provided tumor samples, clinical information, and histopath-ological analysis, ZNW, XZ, YXS, HMX and YZ designed the study and participated in writing the paper. All authors read and approved the manuscript.

## Supplementary Material

Additional file 1 **Supplementary tables**. **Table S1**: RT-PCR primers for amplification of miR-148b. **Table S2**: The sequence of hsa-miR-148b mimics and negative control(NC), anti-miR-148b and anti-NC, siRNA and NC for CCKBR. **Table S3**: Primers used for luciferase reporters construction. **Table S4: **Putative target genes of miR-148b. Click here for file

Additional file 2 **Supplementary figures**. **Figure S1: An overt decrease in the level of miR-148b in ten gastric cancer tissues compared to their matched non-tumor adjacent tissues**. *In situ* detection of miR-148b was performed on paraffin sections using DIG-labeled miRCURY™ Detection probe according to the manufacture's instructions (Exiqon). Images were overlay images with brown color representing miR-148b expression. Obviously, most of the cases revealed a significant decrease in the level of miR-148b in gastric cancer tissues compared to their matched non-tumor adjacent tissues. **Figure S2: Transfection efficiency of miR-148b mimics or anti-miR-148b and their respective NCs and blank controls**. 48 h after transfection, the efficiency of transfection with miR-148b mimics or anti-miR-148b was monitored by qRT-PCR. A, The relative expression of miR-148b which was transfected with miR-148b mimics was very high (476.33 ± 52.97-fold, compared with NC). B, The relative expression of miR-148b which was transfected with anti-miR-148b was significantly low (0.66 ± 0.55-fold, compared with anti-NC). **Figure S3: Apoptosis assay in MGC-803 cells**. For apoptosis assays, floating and adherent cells were harvested 24 h or 48 h after transfection, and then combined and washed with PBS. Annexin-V in combination with propidium iodide (KeyGen) was added to the cells and samples were analyzed within 30 min after staining. The apoptosis assay revealed that miR-148b had no effect on apoptosis in MGC-803 cells. **Figure S4: Transfection efficiency of siRNA and NC for CCKBR. **48 h after transfection, the efficiency of transfection was monitored by qRT-PCR (A) and western blot (B). Click here for file
