# Supplementary material for: MicroRNA-148b is frequently down-regulated in gastric cancer and acts as a tumor suppressor by inhibiting cell proliferation
Source: Mol Cancer. 2011 Jan 4;10:1. doi: 10.1186/1476-4598-10-1 (PMC3024301; doi:10.1186/1476-4598-10-1)
Supplement: Additional file 1 — Supplementary tables. Table S1: RT-PCR primers for amplification of miR-148b. Table S2: The sequence of hsa-miR-148b mimics and negative control(NC), anti-miR-148b and anti-NC, siRNA and NC for CCKBR. Table S3: Primers used for luciferase reporters construction. Table S4: Putative target genes of miR-148b. [file 1476-4598-10-1-S1.PDF]

Additional file 1, Table S1

RT-PCR primers for amplification of miR-148b

	Primer sequence(5’ -3’)
RT—primer1	GCTGTCAACGATACGCTACGTAACGGCATGACAGTGTTTTTTTTTTTTTTTTTTTTTTTTA
RT—primer2	GCTGTCAACGATACGCTACGTAACGGCATGACAGTGTTTTTTTTTTTTTTTTTTTTTTTTC
RT—primer3	GCTGTCAACGATACGCTACGTAACGGCATGACAGTGTTTTTTTTTTTTTTTTTTTTTTTTG
miR-148b-F	TCAGTGCATCACAGAACTTTGTAA
miR-148b-R	GCTGTCAACGATACGCTACGT
U6 RNA-F	CGCTTCGGCAGCACATATAC
U6 RNA-R	TTCACGAATTTGCGTGTCAT

(F) Forward primer; (R) Reverse primer.

Additional file 1, Table S2

The sequence of hsa-miR-148b mimics and negative control(NC) , anti-miR-148b and anti-NC, siRNA and NC for CCKBR

	sequence(5'-3')
hsa-miR-148b mimics	UCAGUGCAUCACAGAACUUUGU<br>AAAGUUCUGUGAUGCACUGAUU
negative control	UUCUCCGAACGUGUCACGUdTdT<br>ACGUGACACGUUCGGAGAAdTdT

	sequence(5'-3')
anti-miR-148b	ACAAAGUUCUGUGAUGCACUGA
anti-NC	CAGUACUUUUGUGUAGUACAA

	sequence(5'-3')
siRNA-CCKBR	AAGCGCGTGGTGCGAATGTTG
NC	AAGCTTCATAAGGCGCATAGC

Additional file 1, Table S3

Primers used for luciferase reporters construction.

	Primer sequence(5’ -3’)
CCKBR F	GCTCTAGAGGCAAATGACATGCACTGAC
CCKBR R	GCTCTAGAACTGGAAAGGGATGAAGAAC
DNMT1 F	GCTCTAGATTCTGCCCTCCCGTC
DNMT1 R	GCTCTAGAGGTTTATAGGAGAGATTTATTTG
WNT10B F	GCTCTAGAGCCCTTTGCTCTGATTTCCT
WNT10B R	GCTCTAGACCCAGCCAAAAGGAGTATGA
ROBO1 F	GCTCTAGACCTGTCTTAACTGGCCTAAA
ROBO1 R	GCTCTAGAGAAAGCAAGTAATGCCTCTA
NOG F	GCTCTAGAGTTCCACCACCCTCTAGC
NOG R	GCTCTAGATAAATTAAAACTGGGACCGT
CCKBR-conserved F	GCTCTAGA ACAGGACTGATTCTGGGATG
CCKBR-conserved R	GCTCTAGAACTGGAAAGGGATGAAGAAC
CCKBR-poorly conserved F	GCTCTAGAGGCAAATGACATGCACTGAC
CCKBR-poorly conserved R	GCTCTAGACAGGCTCAGTCCCATATCAG
Positive control (PC) F	CTAGAACAAAGTTCTGTGATGCACTGAT
Positive control (PC) R	CTAGATCAGTGCATCACAGAACTTTGTT

(F) Forward primer; (R) Reverse primer.

Additional file 1, Table S4

Putative target genes of miR-148b

Genbank No.	Official symbol	Official full name
NM_176875	CCKBR	cholecystokinin B receptor
NM_001130823	DNMT1	DNA (cytosine-5-)-methyltransferase 1
NM_001924	GADD45A	growth arrest and DNA-damage-inducible, alpha
NM_005450	NOG	noggin
NM_002941	ROBO1	roundabout, axon guidance receptor, homolog 1 (Drosophila)
NM_003394	WNT10B	wingless-type MMTV integration site family, member 10B
NM_020156	C1GALT1	core 1 synthase, glycoprotein-N- acetylgalactos-amine 3-beta-galactosyltransferase, 1
NM_003895	SYNJ1	synaptojanin 1
NM_004299	ABCB7	ATP-binding cassette, sub-family B (MDR/TAP), member 7
NM_033150	COL2A1	collagen, type II, alpha 1
NM_016203	PRKAG2	protein kinase, AMP-activated, gamma 2 non-catalytic subunit

The miRNA targets predicted by computer-aided algorithms were obtained from PicTar, http:// pictar.bio.nyu.edu, TargetScan, http://www.targetscan.org , and miRBase Targets, http://www.microrna.sanger.ac.uk. Then, the overlap of these results were further studied by Expression Analysis Systematic Explorer (EASE) analysis based on Gene ontology database and KEGG pathway database.
