# Supplementary material for: MicroRNA-148b is frequently down-regulated in gastric cancer and acts as a tumor suppressor by inhibiting cell proliferation
Source: Mol Cancer. 2011 Jan 4;10:1. doi: 10.1186/1476-4598-10-1 (PMC3024301; doi:10.1186/1476-4598-10-1)

Additional file 2, Figure S1

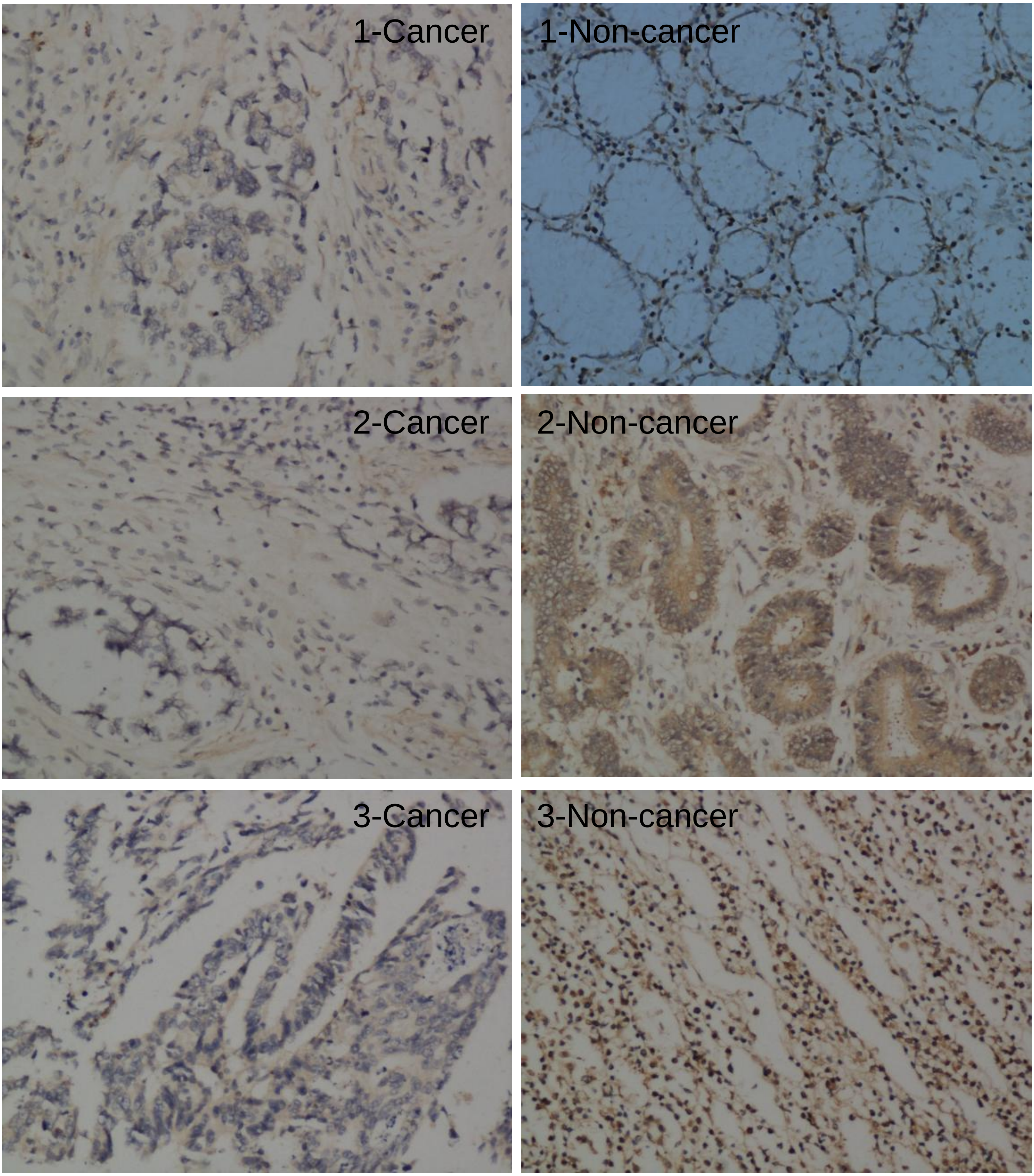

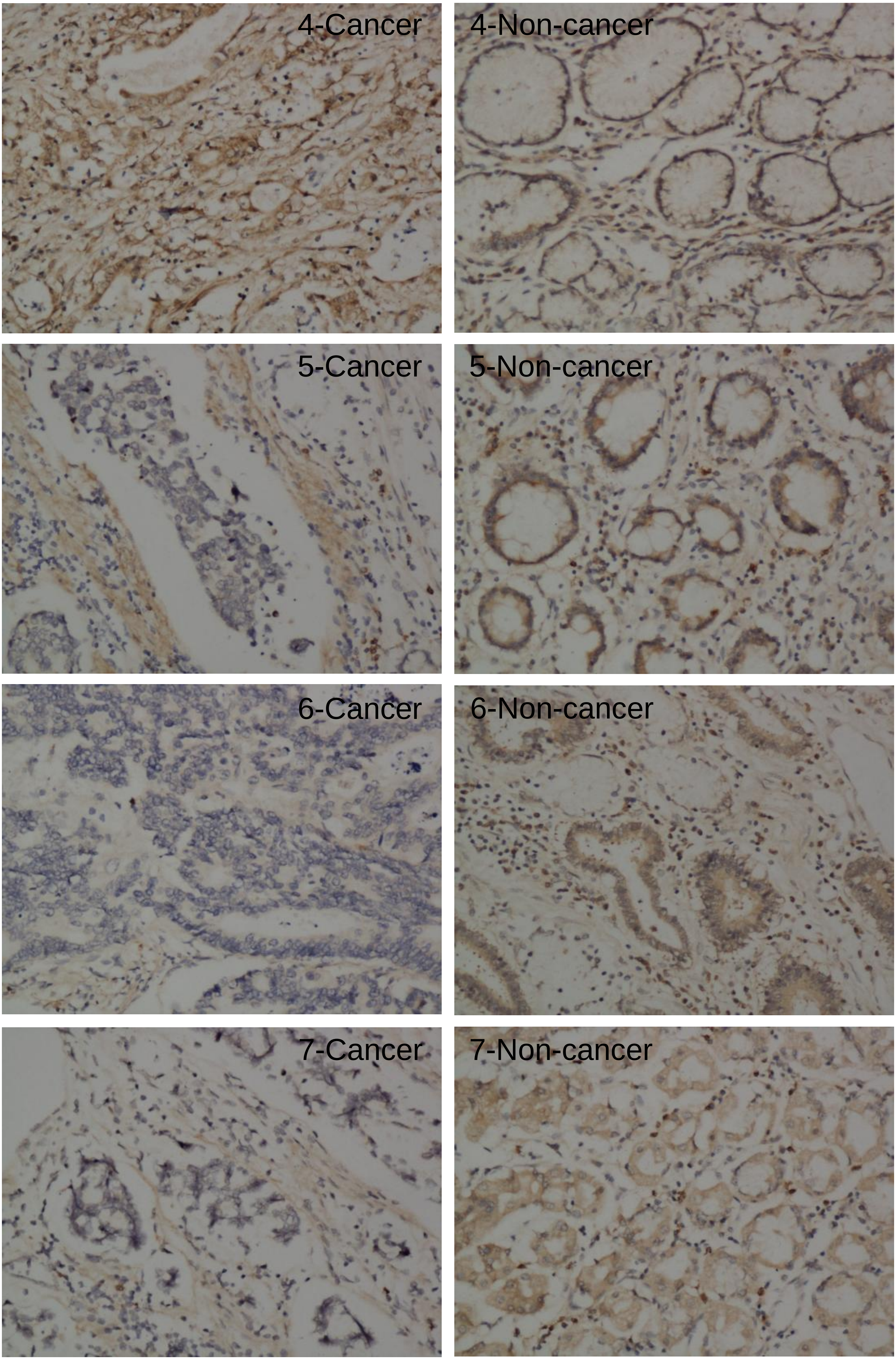
4-Cancer
4-Non-cancer
5-Cancer
5-Non-cancer
6-Cancer
6-Non-cancer
7-Cancer
7-Non-cancer

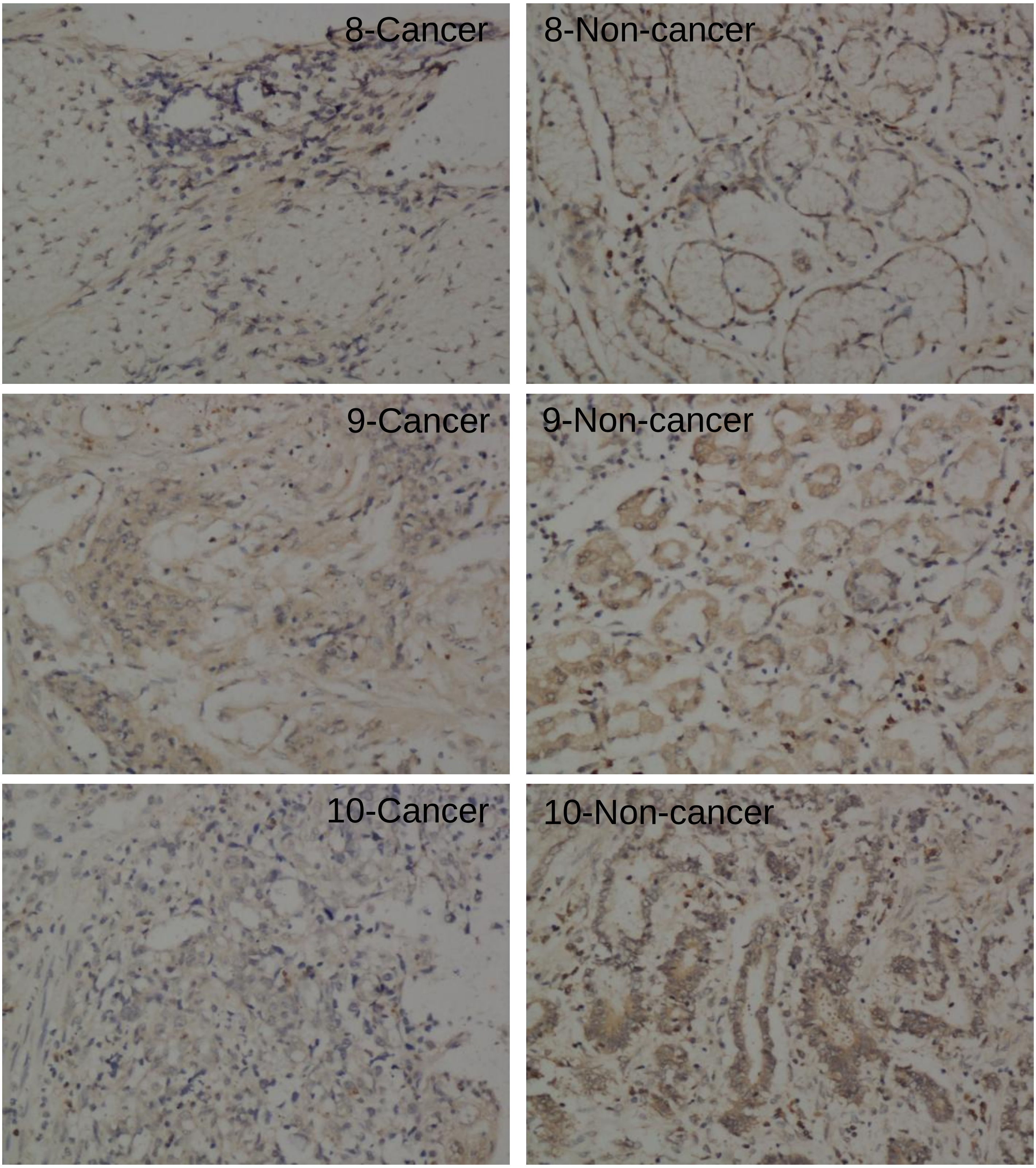
8-Cancer
8-Non-cancer
9-Cancer
9-Non-cancer
10-Cancer
10-Non-cancer

Additional file 2, Figure S2

A

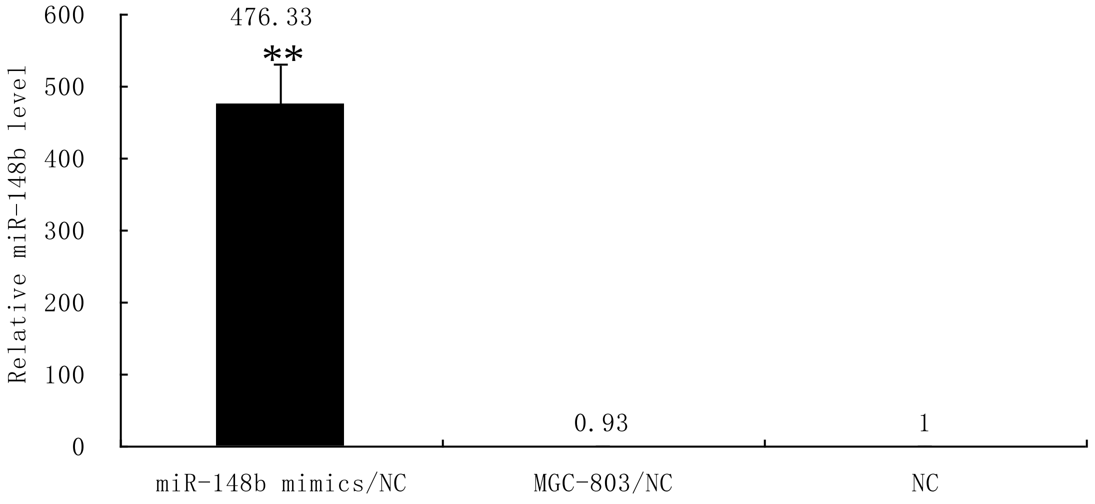

B

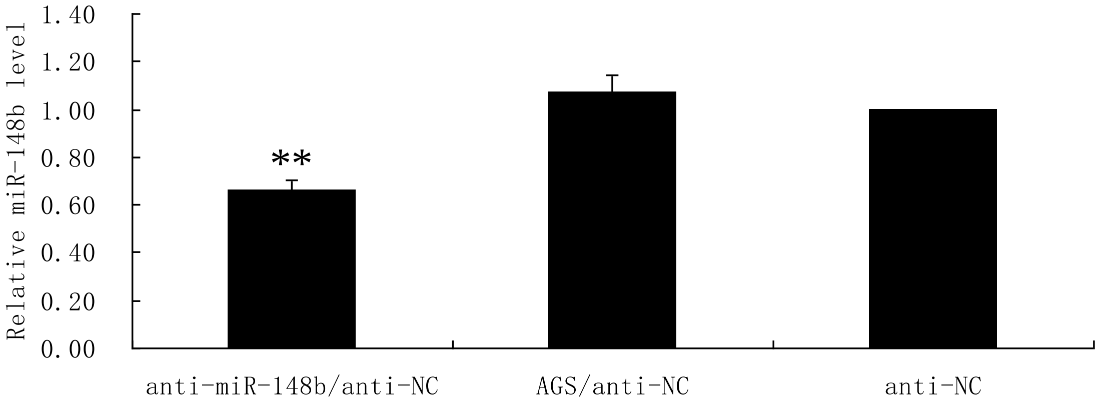

Additional file 2, Figure S3

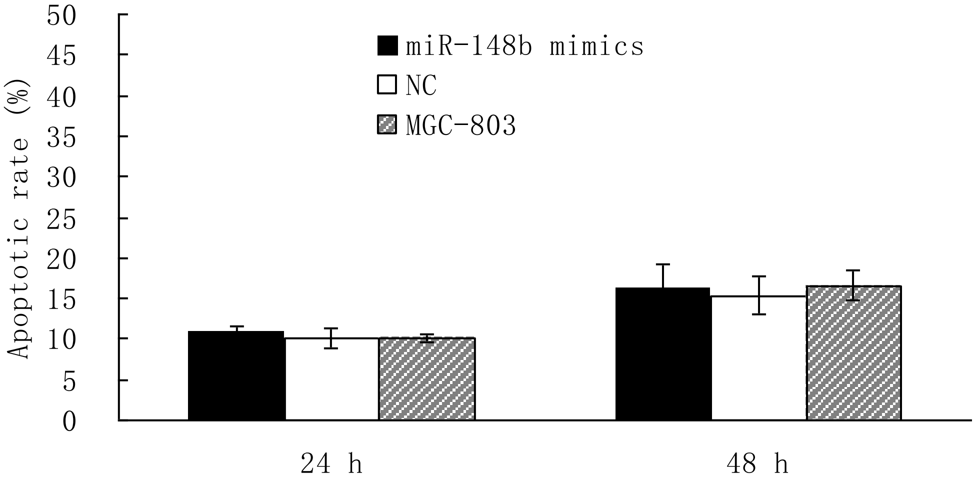

Additional file 2, Figure S4

A

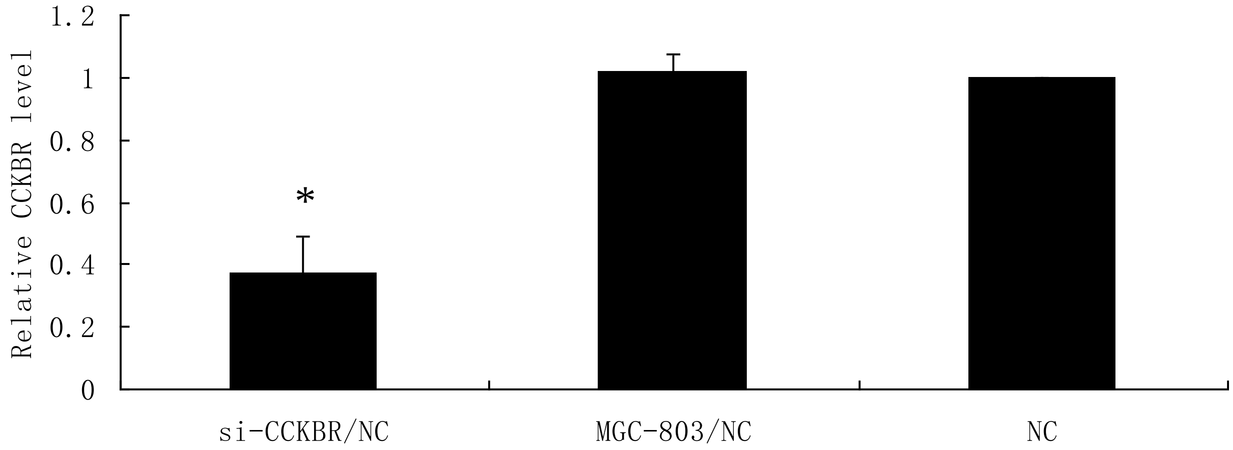

B

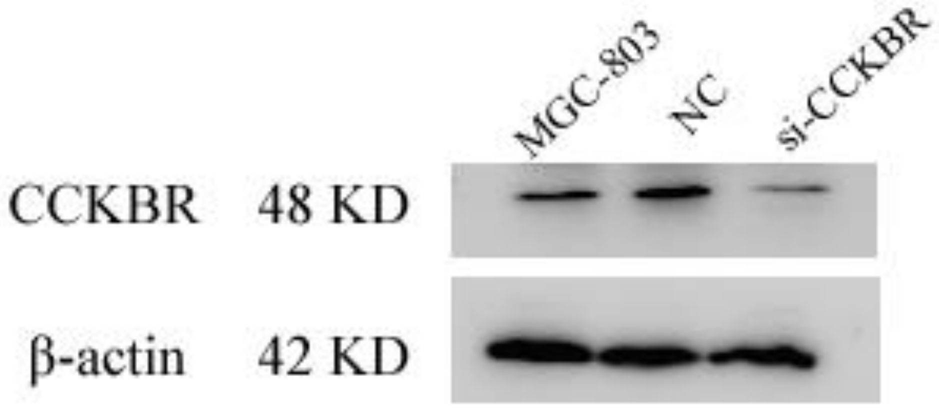

Supplement: Additional file 2 — Supplementary figures. Figure S1: An overt decrease in the level of miR-148b in ten gastric cancer tissues compared to their matched non-tumor adjacent tissues. In situ detection of miR-148b was performed on paraffin sections using DIG-labeled miRCURY™ Detection probe according to the manufacture's instructions (Exiqon). Images were overlay images with brown color representing miR-148b expression. Obviously, most of the cases revealed a significant decrease in the level of miR-148b in gastric cancer tissues compared to their matched non-tumor adjacent tissues. Figure S2: Transfection efficiency of miR-148b mimics or anti-miR-148b and their respective NCs and blank controls. 48 h after transfection, the efficiency of transfection with miR-148b mimics or anti-miR-148b was monitored by qRT-PCR. A, The relative expression of miR-148b which was transfected with miR-148b mimics was very high (476.33 ± 52.97-fold, compared with NC). B, The relative expression of miR-148b which was transfected with anti-miR-148b was significantly low (0.66 ± 0.55-fold, compared with anti-NC). Figure S3: Apoptosis assay in MGC-803 cells. For apoptosis assays, floating and adherent cells were harvested 24 h or 48 h after transfection, and then combined and washed with PBS. Annexin-V in combination with propidium iodide (KeyGen) was added to the cells and samples were analyzed within 30 min after staining. The apoptosis assay revealed that miR-148b had no effect on apoptosis in MGC-803 cells. Figure S4: Transfection efficiency of siRNA and NC for CCKBR. 48 h after transfection, the efficiency of transfection was monitored by qRT-PCR (A) and western blot (B). [file 1476-4598-10-1-S2.PDF]
